# The physics of dancing peanuts in beer

**DOI:** 10.1098/rsos.230376

**Published:** 2023-06-14

**Authors:** Luiz Pereira, Fabian B. Wadsworth, Jérémie Vasseur, Markus Schmid, Simon Thivet, Rafael B. Nuernberg, Donald B. Dingwell

**Affiliations:** ^1^ Department of Earth and Environmental Sciences, Ludwig-Maximilians-Universität München, Munich 80333, Germany; ^2^ Department of Earth Sciences, Durham University, Durham DH1 3LE, UK; ^3^ Institut Charles Gerhardt Montpellier, Université de Montpellier, Montpellier 34293, France

**Keywords:** bubbles, nucleation, floatation, Stokes settling, multiphase system

## Abstract

In Argentina, some people add peanuts to their beer. Once immersed, the peanuts initially sink part way down into the beer before bubbles nucleate and grow on the peanut surfaces and remain attached. The peanuts move up and down within the beer glass in many repeating cycles. In this work, we propose a physical description of this dancing peanuts spectacle. We break down the problem into component physical phenomena, providing empirical constraint of each: (i) heterogeneous bubble nucleation occurs on peanut surfaces and this is energetically preferential to nucleation on the beer glass surfaces; (ii) peanuts enshrouded in attached bubbles are positively buoyant in beer above a critical attached gas volume; (iii) at the beer top surface, bubbles detach and pop, facilitated by peanut rotations and rearrangements; (iv) peanuts containing fewer bubbles are then negatively buoyant in beer and sink; and (v) the process repeats so long as the beer remains sufficiently supersaturated in the gas phase for continued nucleation. We used laboratory experiments and calculations to support this description, including constraint of the densities and wetting properties of the beer–gas–peanut system. We draw analogies between this peanut dance cyclicity and industrial and natural processes of wide interest, ultimately concluding that this bar-side phenomenon can be a vehicle for understanding more complex, applied systems of general interest and utility.

## 1. Introduction

In Argentina, some people add a few (*ca* 10) roasted, shelled and unbroken peanuts (*Arachis hypogaea*) to lager-style beers [1,2]. Peanuts are denser than the liquid beer and so the expectation might be that they should sink to the bottom of the glass. However, when immersed, peanuts do not sink completely, and instead they present a peculiar behaviour: continuous movement up and down. This is called the ‘peanut dance’, the origin of which is thought to be related to the bubbles in a freshly poured beer; however, the detailed dynamics have not been described [1]. In this work, we break down the peanut dancing behaviour into component physical phenomena, which we describe in turn.

**Figure 1.**
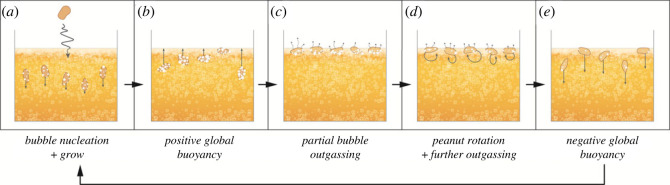
A scheme for the dancing peanut cyclicity in beer. (*a*) Peanuts are introduced into the beer, sink part way and work as nucleation sites for bubbles; (*b*) bubble–peanut aggregates rise due to positive buoyancy; (*c*) bubbles are released by bursting at the free surface; (*d*) peanuts rotate on the free surface allowing further outgassing; (*e*) bubble–peanut aggregates become negatively buoyant and sink.

Phenomenologically, when roasted, shelled and unbroken peanuts are first introduced to a glass of lager-style beer, they sink. During the initial sinking phase, they act as a nucleation site for bubbles (figure 1*a*), because only a few seconds after immersion, the surface of the peanut is covered with bubbles. It can be observed that those bubbles covering the peanut do not originate from rising bubbles from below. The nucleated bubbles remain attached to the peanut surfaces and start to grow; at a certain point, the bubble-coated peanut reverses direction and starts to float (figure 1*b*). Once the bubble-coated peanut reaches the beer top surface, some (but not all) of the bubbles attached to the top part of the peanut and proximal to the atmosphere or beer foam are released by bubble bursting (figure 1*c*). After some time at the top surface, the peanut rolls and rotates, which allows bubbles on the underside to also detach and pop at the beer top surface (figure 1*d*). The peanut–gas assembly then sinks into the body of the beer, and the cycle can repeat via new preferential nucleation on the peanut surfaces as well as by growth of any remaining attached bubbles (figure 1*e*).

Here, we present constraints of the physical properties of a three-phase (beer–gas–peanut) system and calculations associated with the preferential bubble nucleation on the peanut surface as well as the system dynamics. The latter allows us to estimate the critical number and size of bubbles required to reverse the buoyancy from sinking to floating, and back again. Ultimately, like in any good bar-side conversation among scientists, we use the peanut dance to discuss similar processes occurring in other fields from industrial to natural processes.

## 2. Material characterization and methods

The phenomena introduced here (figure 1) suggest that the processes relevant to the peanut dance dynamics are broadly going to fall into three categories: (i) bubble nucleation, (ii) buoyancy effects and (iii) cyclicity. Therefore, we surmise that the wetting properties of beer–gas–peanut and the component densities are going to be crucial properties of the system. Here, we either present these data from published literature, or, where specific determinations are not available, we measure the properties. The collated values were obtained for lager-style beer and roasted-shelled peanuts (*Arachis hypogaea*). Lager-style beers generally contain around 92–96 wt.% water, resulting in a density very similar to water [3]. This beer density value was obtained from ‘JavaScript beer specs calculator’ [4] (*ρ*_beer_ = 1012 kg m^−3^) which is a value similar to the one found in Liger-Belair & Cilindre [5]. Beer-vapour surface tension was also retrieved from the literature (*σ*_beer_ = 41.55 ± 1.39 mN m^−1^) [6]. Bubbles in most beers are composed of carbon dioxide gas and can be assumed to be at 1 bar pressure (atmospheric) once the beer is poured (*ρ*_bubble_ = 1.98 kg m^−3^).

Roasted-shelled peanut density (*ρ*_peanut_) measurements were performed here at room temperature using the Archimedean method with water as an immersion fluid and a Sartorius LA 230P scale [7]. They were carried out three times per batch of peanuts (*ca* 40 nuts). The final density value was obtained by division of the mass of peanuts by the volume of the displaced liquid a few seconds after immersion. The peanut density at 25°C was found to be *ρ*_peanut_ = 1092.97 ± 18.35 kg m^−3^.

**Figure 2.**
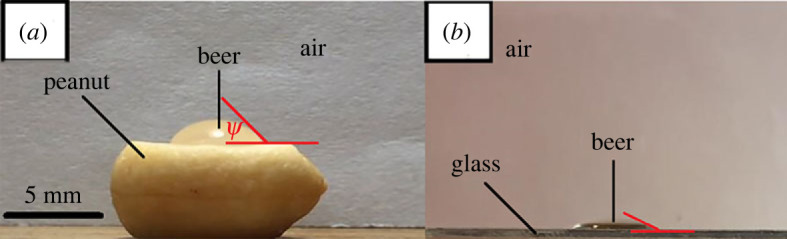
Example of a measurement of contact angle *Ψ* between (*a*) peanut–beer immersed in air and (*b*) glass–beer immersed in air. The average values of 10 measurements are *Ψ*_peanut_ = 46.48 **±** 2.43° and *Ψ*_glass_ = 23.20 **±** 2.80°.

Contact angle (*Ψ*) measurements between the involved phases (solid–liquid–gas) were carried out using the sessile drop method [8]. This method is used to make direct measurements of the contact angle to determine preferential wetting of a given solid by a liquid droplet surrounded by a gas (figure 2). The measurements were performed 10 times for each of the following sets: peanut–beer–air and glass–beer–air. Here, we simplified the analyses using air even though lager beers normally are composed of CO_2_. The contact angle values are respectively *Ψ*_peanut_ = 46.48 ± 2.43° and *Ψ*_glass_ = 23.20 ± 2.80° (figure 2), where the subscript refers to the substrate on which the beer droplet was placed. Considering the opposite situation, in which a bubble wets a peanut's surface and is immersed in beer, the contact angle inside the bubble is the supplementary value of *Ψ* [9]; i.e. 180 − *Ψ* degrees.

The complete peanut dancing phenomenon (of 13 peanuts) was investigated in a 100 × 100 × 200 mm-sized tank containing 1 l of lager-style beer. This process was recorded by a Sony™ camcorder model FDR-AX53 Zeiss™ (8.57 megapixels) operating at 25 frames per second. The process was recorded until the peanuts begin to settle to the bottom of the container and the dancing phenomenon stops. This end to the process occurs after approximately 150 min and is associated with the degassing of the beer to the point where bubble nucleation can no longer occur at sufficient rates to stop the peanuts sinking. Image analysis was carried out manually with Fiji and the MTrackJ plugin on the video frames to constrain the size and number of bubbles per peanut as well as the peanut sizes [10]. The different numbers of bubbles per peanut were obtained by multiplying the visible counted value by two. We selected three different time windows to execute the image analyses: 2–13 min, 60–64 min and 120–126 min after the peanut introduction into the beer. Three different videos at these aforementioned time windows are available in the electronic supplementary material as movies S1 (starts at minute 2), S2 (starts at minute 60) and S3 (starts at minute 120). These available movies have a full-HD resolution. The smallest detectable bubble is 50 µm.

## 3. Results and discussion

In this section, the primary results for bubble nucleation, buoyancy and cyclicity are presented to support the findings in order to elucidate the dominant processes. First, we find that the equivalent peanut radius occurs in a range of sizes from a maximum $R_{\mathrm{peanut}}^{\max}=7.16\;\mathrm{mm}$ to a minimum $R_{\mathrm{peanut}}^{\min}=4.88\;\mathrm{mm}$.

### 3.1. Bubble nucleation on peanuts in beer

Beers are stored under modest pressure. Most beers, including lagers, involve CO_2_ as the dominant dissolved gas species [5]. When beers are opened or poured from a tap, they decompress, which induces supersaturation of CO_2_ and bubble nucleation [11–15]. Classical nucleation theory (CNT) [16] predicts homogeneous nucleation in a liquid and considers that nucleation takes place when molecules of the separating phase form a cluster larger than the critical radius. The critical radius (*r*_c_) and the critical free energy for nucleation ($\Delta G_{c}$) are governed by the energy balance between the bulk free energy per unit of volume ($\Delta G_{v}$) and the surface energy per unit of area between the new phase and the surrounding liquid. The nucleating phase has a lower bulk free energy per unit of volume than the supersaturated liquid (negative $\Delta G_{v}$). In the case of bubble nucleation, the term $\Delta G_{v}$ is proportional to the supersaturation pressure of the system (ΔP) (i.e. the difference between the ambient pressure in the liquid and the saturation pressure) [13]. By contrast with homogeneous nucleation, heterogeneous nucleation is facilitated by an external surface and consequently the affinity between the new gaseous phase and the solid surface is of importance [13,15]. Based on CNT, Δ*G*_c_ is

3.1 $$\Delta G_{c}=\frac{16\pi \sigma^{3}}{3\Delta P^{2}}\alpha ,\;$$

where *σ* is the surface tension and *α* is a geometric parameter that is defined as a function of the contact angle *ψ* [14]:

3.2 $$\alpha =\frac{(2-\cos(\psi )){\left(1+\cos(\psi )\right)}^{2}}{4}.$$

In other words, the geometric parameter *α* reflects how much easier it is to nucleate a bubble heterogeneously compared with homogeneously, such that *α* = 1 is the homogeneous nucleation case and $\Delta G_{c}$ reduces to 16*πσ*^3^/(3Δ*P*^2^).

**Figure 3.**
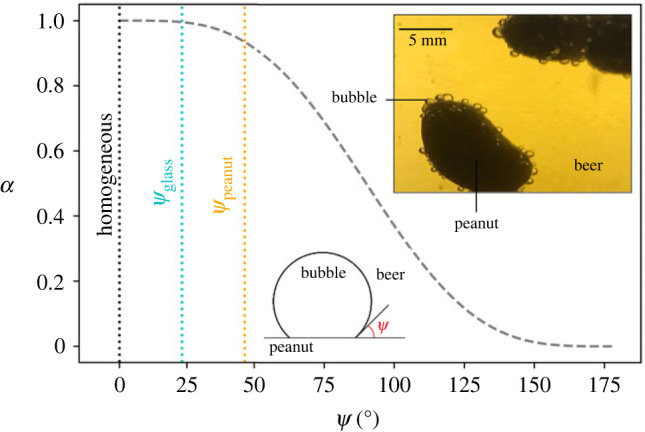
The geometric parameter *α* as a function of the contact angle *ψ*. The dashed curve is given by equation (3.2). Shown here are the three values determined herein for bubbles forming in beer: in the bulk liquid (homogeneous), on clean glass surfaces (*ψ*_glass_) and on roasted-shelled peanut surfaces (*ψ*_peanut_); the latter being the larger *ψ* and therefore the lower value of α. Inset is a photograph of a peanut immersed in beer, showing the high relative contact angle *ψ* for nucleation.

In the system considered herein, there may be three possible ways to nucleate a bubble: on the peanut (heterogeneous), on the glass wall (heterogeneous), or in the bulk liquid (homogeneous). In figure 3, we show the geometric parameter *α* as a function of the contact angle *ψ*. The geometric parameter for the peanut–beer–gas system is the smallest one, followed by the one of the glass–beer–gas, and finally the homogeneous nucleation case (*α* = 1). It can be observed from equation (3.1) that the smaller the *α* term, the lower the energy required for bubble nucleation. Hence, through equations (3.1) and (3.2) along with figure 3, we conclude that heterogeneous nucleation on a peanut is energetically favourable, followed by nucleation on the glass wall. The least favourable situation is homogeneous nucleation in the body of the beer. In figure 3, by way of illustration, a peanut with several bubbles as well as a schematic drawing of a bubble wetting the peanut's surface are shown. The contact angle displayed in the schematic drawing of figure 3 reflects the measured contact angle value.

The simple CNT introduced here considers that the surface used for bubble nucleation is flat and smooth. The assumption that the surface is flat will hold in the case of peanut–gas–beer systems because the bubble length scales are much smaller than the peanut itself. Our assumption that the surface is smooth may not hold in some situations; however, any surface roughness will only serve to promote nucleation. Therefore, our calculations here are a conservative estimate.

It is important to underline that bubble growth from existing gas cavities on glass requires very low energies and, if the gas cavity volumes are equivalent to a bubble radius that is greater than the critical radius, then there is zero energy barrier [11–13]. This type of bubble formation is thought to be the origin of the so-called ‘bubble-trains’, which are common in sparkling drinks, such as beers and champagne [11,12]. However, only a few ‘bubble-trains’ are typically observed in pristine unscratched beer glasses and therefore the bulk of the liquid is not enough degassed by this mechanism, and peanuts in the bulk of the beer liquid are the most favourable nucleation sites.

### 3.2. Buoyancy

Here, we ask the question: is the bubble–peanut nucleation attachment stable? To answer this question, we follow the approach presented in Gualda & Ghiorso [9]. According to their study, the attachment force is obtained by considering the change in surface energy between the state where the bubble and solid are apart and that where bubble and solid are attached. They define the attachment force between a bubble and a particle (peanut in our case) *F*_att_ as

3.3 $$F_{\mathrm{att}}=2\pi R_{\mathrm{bubble}}\sin^{2}(\psi )\sigma_{\mathrm{beer}}{\left[\frac{4}{2+3\cos(\psi )-\cos^{3}(\psi )}\right]}^{1/3},$$

as well as the total forces for a peanut *F*_peanut_ and for a bubble *F*_bubble_ as

3.4 $$F_{\mathrm{peanut}}=-\frac{4}{3}\pi R_{\mathrm{peanut}}^{3}g(\rho_{\mathrm{peanut}}-\rho_{\mathrm{beer}}),$$

3.5 $$F_{\mathrm{bubble}}=-\frac{4}{3}\pi R_{\mathrm{bubble}}^{3}\beta g(\rho_{\mathrm{bubble}}-\rho_{\mathrm{beer}})$$

3.6 $$\mathrm{and}\;\beta =\frac{1}{2}+\frac{\cos(\psi )}{4}(2+\sin^{2}(\psi )),$$

where *β* is defined as a function of the contact angle *ψ* and represents the fraction of the sphere volume corresponding to the bubble cap attached to the peanut surface, and for our case, it is almost unity (β∼0.93). In equations (3.3)–(3.5), *g* is the gravitational acceleration, *R*_bubble_ and *R*_peanut_ are the bubble and peanut equivalent spherical radii, respectively. Equation (3.5) is similar to the equation proposed by Gualda & Ghiorso [9] for the forces on the bubble, but here instead of using a simplified spherical geometry, we consider the volume of the bubble cap, which is a function of the aforementioned contact angle value as well as of the equivalent spherical bubble radius. However, as just mentioned, for the studied case, this difference is minimal. Stability of a bubble–peanut assembly is defined as when the attachment force is equal to or larger than the forces acting to separate the bubbles from the peanut [9]. The separating force *F*_separating_ is the difference of the aforementioned forces on a peanut and on a bubble:

3.7 $$F_{\mathrm{separating}}=F_{\mathrm{bubble}}-F_{\mathrm{peanut}}.$$

When the bubble–peanut assembly is neutrally buoyant, *F*_peanut_ and *F*_bubble_ are equal in magnitude, but have opposite signs [9]. In this case of neutral buoyancy, we can substitute *F*_peanut_ for − *F*_bubble_ in equation (3.7), giving

3.8 $$F_{\mathrm{separating}}=\frac{8}{3}\pi R_{\mathrm{bubble}}^{3}\beta g(\rho_{\mathrm{bubble}}-\rho_{\mathrm{beer}}).$$

When *F*_att_ > *F*_separating_, bubbles will remain attached to the peanut. Conversely, when *F*_att_ < *F*_separating_, bubbles will detach under the separating forces. Therefore, our critical condition for stability is that *F*_att_ = *F*_separating_. By setting equation (3.3) equal to equation (3.8), one can find the theoretical critical bubble radius for attachment condition of a bubble to a peanut surface, $R_{\mathrm{critical}}^{t}$. For the measured values used here for our system of interest, this theoretical critical radius is equal to $R_{\mathrm{critical}}^{t}=1.34\;\;\mathrm{mm}$.

**Figure 4.**
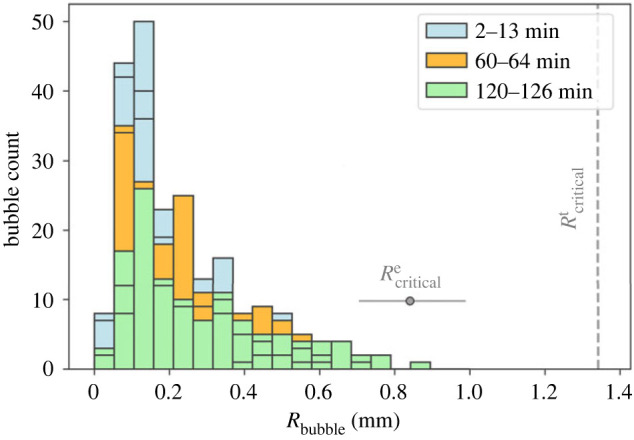
Histogram of bubble radii for experimental data obtained at different time windows: 2–13 min, 60–64 min and 120–126 min after the peanut introduction into the beer. For each window, five peanuts coated with bubbles were analysed. The theoretical critical bubble radius for stable attachment is displayed in the plot as a grey dashed line. The mean and s.d. of bubble radii just before detachment, obtained from image analysis of the electronic supplementary material, are displayed in red.

In figure 4, we show histograms for observed bubble radii in the three time windows of video analysis: 2–13 min, 60–64 min and 120–126 min. We compare these experimental values with the theoretical critical bubble radius for stable bubble–peanut attachment (dashed grey line). This figure also displays the average and the standard deviation of 15 bubble radii measured instants before the detachment events ($R_{\mathrm{critical}}^{e}$). These values were obtained for bubbles in which the detachment was caused by the difference of buoyancy opposed to the detachment of bubbles at the interface between beer and atmosphere. It is interesting to note that early in the experiment (2–13 min) bubbles are generally smaller than at later stages (e.g. 120–126 min) and the size distribution broadens with time due to mass transfer of dissolved carbon dioxide into bubbles. In all cases, we predict that bubble–peanut attachment is stable (figure 4). Furthermore, we point out that bubbles detaching from the peanuts are generally larger than bubbles detaching from the glass walls due to surface effects that make them remain attached to larger sizes on peanuts (see electronic supplementary material, movies S1–S3). These observational results corroborate the simple force balance calculation provided here since all experimental bubble size falls below the theoretical critical radius estimated.

**Figure 5.**
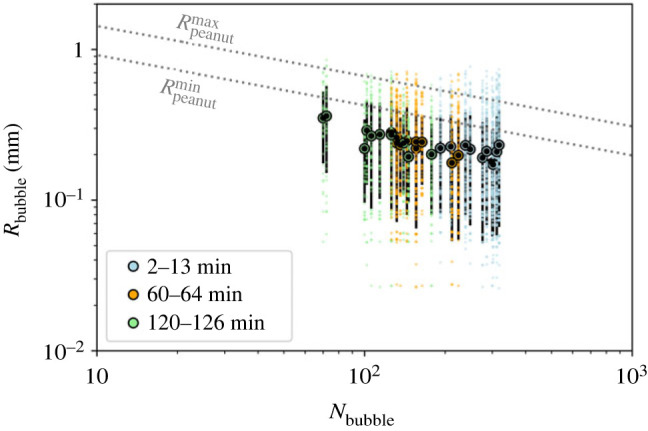
Bubble radius as a function of the number of bubbles per peanut to make the assembly neutrally buoyant (grey lines). The experimental data obtained at different time windows: 2–13 min, 60–64 min and 120–126 min after the peanut introduction into the beer are displayed in as circles (mean), the s.d. as black bars and the whole dataset as little coloured circles.

The next question we address is what is the critical number of bubbles per peanut and their size to make the peanut dance? For this purpose, we rely on the end-member peanut radius values obtained by image analyses of the video frames. For this analysis, equation (3.5) is multiplied by *N*_bubble_ in order to consider the total number of bubbles attached to a single peanut. Thus, by combining equations (3.4), (3.5) and (3.6) along with these peanut end-member values, we obtain curves describing the limiting values for neutrally buoyant situations as functions of *N*_bubble_ and *R*_bubble_ values (figure 5). Above these lines the system is positively buoyant and peanut floating occurs, whereas below, the systems are negatively buoyant and peanut sinking occurs. We highlight the mean value (black circles) for each situation as well as the s.d. (black bars) and all the measured bubble radius values (coloured points). There are experimental results for negative, neutral, and positive buoyancy, which characterize the dancing phenomenon. Despite the fact that the average is below the ‘dancing’ range, if one considers the s.d., it can be noticed that the system falls in the zone where the dance occurs. Also, the small discrepancy between the average of experimental values and theoretical calculations can be attributed to the cut-off limit of the image analysis procedure (smallest detected bubble is 50 µm). It can be observed that the bubbles grow with time and that at longer times, there are fewer bubbles per peanut.

We note that the bubble–peanut–beer system exhibits properties (densities, contact angle, peanut sizes, number of bubbles per solid particle) that are just right for the dancing spectacle to be physically feasible and therefore observed.

### 3.3. Cyclicity

**Figure 6.**
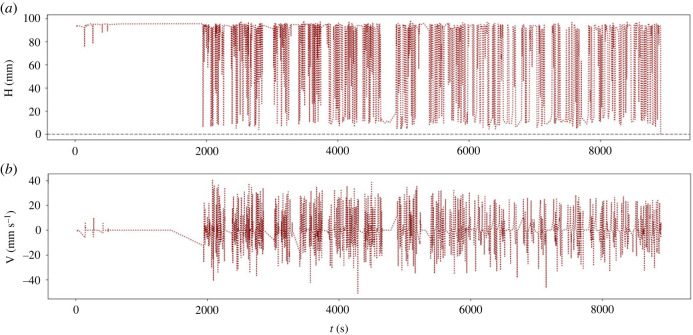
Quantitative observations of the peanut dance. The upper plot displays the peanut vertical position as a function of time, and the bottom one, the peanut velocity as a function of time.

The dancing peanuts phenomenon is cyclic. In our image analysis steps, we tracked peanut position during the entire dancing peanut lifetime, i.e. approximately 150 min long for the studied case (figure 6). We observe that in the initial stages, the bubble nucleation rate, which is closely related to the supersaturation of gas, is high relative to the later stages [15]. The elevated relative bubble nucleation rate may be higher than the bubble detachment rate at the top surface, which acts to cause peanuts to congregate at the top surface of the glass of beer. After some time (approx. 25–30 min), the peanuts start to cycle up and down, creating the dancing phenomenon. The frequency of the up–down cycles becomes larger in the intermediate stages of the whole process and then wanes to zero toward the end. This waning is associated with the drop in gas supersaturation. This results in peanuts that come to rest on the bottom of the glass for some time. In a related manner, we observe that the maximum speed of the peanuts decreases with time. These behaviours can be observed in figure 6. It is interesting to observe that terminal velocities of the bubble–peanut assembly are not reached in our system (figure 6). This is because the bubble nucleation rate on the peanut surface is too high with respect to the vertical distance travelled by the peanut–bubble assembly to allow the system to reach a terminal velocity.

## 4. Similar processes in industrial and natural systems

Like any good bar-side conversation starter, the peanut dance is a vehicle for discussions about particle floatation in general. To give one example: Moinester *et al*. [17] studied a circular sweet (fizz-ball) immersed in sparkling water. The fizz-ball undergoes up–down cycles similar to those observed for the dancing peanuts. Here, we survey similar systems, in industrial and natural contexts, with comparable floatation problems for which the peanuts-in-beer may be a model system.

### 4.1. Froth floatation

Froth floatation is a physico-chemical process used to separate different particle populations in liquids based on differences in surface wettability [18]. It consists of the introduction of bubbles in a liquid (generally water) containing different types of particles whereby, due to the difference of wettability of these entities, the floatation of a select particle type takes place. Just as with the peanuts in beer, the bubble–particle attachment and contact angle values are the key factors behind this floatation process [9,18,19]. This process is used widely in large-scale facilities in mining, where ore products are crushed to micrometric particles and then mixed with water to form a pulp. Air bubbles are blown into this system in a controlled way to force the desired mineral particles to bind to them due to their surface affinity (cf. figure 2). The froth floatation process is designed in such a way as to enable the bubble–particle aggregates to rise to the upper surface, where they are collected [18]. The gangue (the commercially undesirable remnant material) generally settles. Froth floatation is also widely used to remove unwanted material for water purification and to clean industrial waste products [20]. Unlike the peanut–beer system, in froth floatation air bubbles are not nucleated due to gas supersaturation, but are introduced to the system [18].

### 4.2. Simplified nuclear waste vitrification system

Glasses are non-crystalline and as a consequence they can incorporate a wide range of elements in their structure [21–23]. This feature, along with their chemical durability and irradiation resistance, makes glass the current choice for immobilizing high-level waste (HLW) materials [21]. Borosilicate compositions are used as a glass matrix for HLW conditioning [24]. By comparing a simplified laboratory-scale system in which borosilicate melt contains ruthenium(IV) oxide crystals (approx. 2.0 vol.%) with the equivalent system without crystals, it has been noticed that RuO_2_ crystals change the dynamics of bubbles [25,26]. Different from other oxides coming from the nuclear waste, RuO_2_ when added to the borosilicate glass matrix during the immobilization procedure, is present as suspended crystals due to its sparing solubility in this type of melt [27]. Similar to the peanut–beer system, despite the appreciable density of these suspended RuO_2_ crystals (6970 kg m^−3^) relative to the borosilicate liquid (2283 kg m^−3^), the entrainment of bubbles carried crystals to the upper surface of the liquid [25,28]. Subsequent bubble outgassing was followed by crystal sinking, generating a cyclic gas-release phenomenon [25]. This mechanism observed in a very simplified laboratory experiment in nuclear waste vitrification context is very similar to the one observed in the dancing peanuts in beers.

### 4.3. Magnetite floatation in magmatic liquids

Magmas in the Earth's crust crystallize over long periods of geologic time. In most cases, the crystals are denser than the liquids from which they form, such that they should sink over time. Magnetite (Fe_3_O_4_) is a mineral that is denser (5150 kg m^−3^) than magmatic liquids (approx. 2500 kg m^−3^) and is expected to settle out and accumulate at the bottom of magma storage regions in the Earth's crust. However, where such systems have undergone complete crystallization, it has often been observed that magnetite-rich regions have been found in the upper part of these systems, and not the lower regions as might be expected due to its greater density [29–31]. Different processes have been invoked to explain this observation. One of the leading hypotheses is that this occurs because of preferential wetting of magnetite by low-density fluids (e.g. exsolved magmatic gas bubbles), followed by buoyant segregation of these magnetite–bubble aggregates [32]. In magmas supersaturated with a volatile gas phase, bubble formation occurs preferentially by heterogeneous nucleation due to its characteristic of reducing the required activation energy value [14] (figure 3). Magnetite has a particularly high contact angle (figure 2), suggesting a particular affinity between magnetite and the volatile gas phases [33–37]. It has been confirmed experimentally that microlites of iron–titanium oxides similar to magnetite exert a larger influence on bubble nucleation than other mineral phases. As the bubbles are nucleated on these iron-rich mineral surfaces, the mineral coated with bubbles tends to become positively buoyant in the silicate system [32]. Gualda & Ghiorso [9] demonstrate that this magnetite–bubble attachment for relevant magmatic scenarios was indeed feasible and could result in rising dense crystals. Knipping *et al*. [38] experimentally tested the hypothesis that bubbles entrain magnetite crystals to the upper part of the crustal storage system.

Further in geoscience, Mungall *et al*. [39] demonstrate that floatation of metals and sulfur attached to vapour bubbles is a plausible mechanism to explain the upward mobility of sulfide liquids to the shallow crust. Similarly, Iacono-Marziano *et al*. [40] have recently revealed the role of volatile-containing bubbles in the formation of magmatic sulfide deposits, which are an important source of platinum-group elements, nickel, copper and cobalt. These authors demonstrated how the association between sulfide droplets and bubbles may facilitate the coalescence of sulfide droplets and ultimately culminate in the upgrading of metal content in the sulfide liquid phase.

## 5. Conclusion and outlook

We have studied a dancing peanut phenomenon in degassing lager-style beers. We performed a series of laboratory measurements and calculations that have allowed us to break down the problem of the dancing peanuts into different physical phenomena. Based on these experimental and theoretical approaches, we arrive at a framework centred on the preferential heterogeneous nucleation of stable attached bubbles that cause peanuts to become buoyant. This is followed by a rolling outgassing at the beer free surface, before subsequent peanut sinking and repetition of the process. Clearly then, the conclusion is that the period of the dancing motion is sensitive to the nucleation rate, to the detachment rate at the upper surface, and to bubble sizes on the peanuts, which in turn will be different in different beer varieties. Similarly, different peanut types—salted, roasted, shelled, etc.—could have substantial controls on the dynamics via changes in wettability or density. We survey some similar systems in silicate glasses and magmas. Dynamic similarities between beer and natural silicates have been proposed previously: Manga [41] proposed a key similarity between bubble-bearing magma dynamics and bubbles in Guinness. Therefore, we close by proposing that this study has heritage and that the observation of bubble dynamics in beer is a rich topic, worth repeated investigation.

## Data Availability

The data are available from the Dryad Digital Repository: https://doi.org/10.5061/dryad.xsj3tx9m0 [42]. Supplementary material is available online [43].
